# The Use of Hydrogels for the Treatment of Bone Osteosarcoma via Localized Drug-Delivery and Tissue Regeneration: A Narrative Review

**DOI:** 10.3390/gels9040274

**Published:** 2023-03-25

**Authors:** Shebin Tharakan, Iman Raja, Annette Pietraru, Elina Sarecha, Andrei Gresita, Eugen Petcu, Azhar Ilyas, Michael Hadjiargyrou

**Affiliations:** 1 College of Osteopathic Medicine, New York Institute of Technology, Old Westbury, NY 11568, USAiraja@nyit.edu (I.R.); agresita@nyit.edu (A.G.);; 2 Department of Biological and Chemical Sciences, New York Institute of Technology, Old Westbury, NY 11568, USA; 3 Department of Electrical and Computing Engineering, New York Institute of Technology, Old Westbury, NY 11568, USA

**Keywords:** osteosarcoma, hydrogels, bone, scaffold, tissue engineering, drug-delivery

## Abstract

Osteosarcoma is a malignant tumor of bone that leads to poor mortality and morbidity. Management of this cancer through conventional methods involves invasive treatment options that place patients at an increased risk of adverse events. The use of hydrogels to target osteosarcoma has shown promising results both in vitro and in vivo to eradicate tumor cells while promoting bone regeneration. The loading of hydrogels with chemotherapeutic drugs provides a route for site-specific targeted therapy for osteosarcoma. Current studies demonstrate tumor regression in vivo and lysis of tumor cells in vitro when exposed to doped hydrogel scaffolds. Additionally, novel stimuli-responsive hydrogels are able to react with the tissue microenvironment to facilitate the controlled release of anti-tumor drugs and with biomechanical properties that can be modulated. This narrative review of the current literature discusses both in vitro and in vivo studies of different hydrogels, including stimuli-responsive, designed to treat bone osteosarcoma. Future applications to address patient treatment for this bone cancer are also discussed.

## 1. Introduction

Bone malignancies are an uncommon sporadic form of cancer primarily affecting adolescents and older adults. Primary bone tumors are rare and account for less than 1% of all cancers. Rather, bone metastasis is a commonly seen phenomenon, with 30–75% of advanced malignant tumors presenting this way [1]. Of the primary bone tumors, osteosarcoma is the most common primary bone cancer overall in children and teens; however, it can also be seen in the elderly [2]. In adults, the most common primary bone tumor is chondrosarcoma, followed by chordoma and osteosarcoma [3]. Osteosarcoma has the lowest 5-year survival rate for bone cancers across all Surveillance, Epidemiology, and End Results (SEER) stages. Osteosarcoma has a 60% 5-year survival rate compared to 79% in chondrosarcoma and giant cell tumor of bone [4]. Osteosarcoma takes a severe toll on patients by a reduction of their quality of life, even with or without treatment.

Unfortunately, osteosarcoma is a difficult tumor to treat. Often, physicians recommend surgery, radiation, chemotherapy, or a combination of these options as aggressive treatment. Currently, neoadjuvant chemotherapy with surgical resection of the tumor, followed by additional adjuvant therapy, is one of the current regimens for osteosarcoma therapy [5]. In the past, severe osteosarcoma would require the removal of a large portion of bone, often resulting in limb amputation [6,7]. With the advancement of surgical precision and techniques, patients can undergo limb-sparing surgery with a prosthetic replacement or graft [8,9,10]. While nearly 85% of osteosarcomas are treated this way, surgery is highly invasive, and patients endure a loss of function in mobility and lifestyle [11]. The diagnosis, treatment, and management of osteosarcoma place a physical and psychological burden on patients and their families and a financial burden on the healthcare system. Additionally, surgical complications may predispose patients to infection, periprosthetic fractures, and dehiscence [12]. Coupled with the enormous side-effect profile of chemotherapeutic agents, patients often tolerate the treatment very poorly despite increased survival [5,13].

Current in vivo and in vitro studies focus on generating mouse models to identify and target ‘driver mutations’ for effective drug therapy. Numerous gene clusters have been identified to contribute to osteosarcoma: p53, Notch1, PTEN, BRCA2, FOS, RET, ATM, and FANCA amongst others [14]. Driver-dependent therapy targets constitutively active signaling pathways to downregulate tumor growth. In osteosarcoma, the PI3K/mTOR signaling pathway has been identified as common for cancer proliferation [15]. Unfortunately, in nearly 25% of patients, this altered pathway is affected, thus, limiting kinase-targeted treatment to just this subset of patients [15]. However, the most commonly seen mutation is in p53, but therapeutic targeting of p53 is difficult due to many mutations and downstream effects [14]. Investigation into the pharmaceutical modulation of p53 has been conducted with Nutlin-3a, a ubiquitin ligase antagonist (MDM2 antagonist) [16]. Preclinical sensitivity to osteosarcoma cells was observed; however, the clinical activity in patients with liposarcoma indicated an unsatisfactory response [17]. Novel MDM2 inhibitors have not been tested in treating osteosarcoma in preclinical or clinical settings. Therefore, current chemotherapy focuses on using conventional chemotherapeutic drugs such as methotrexate, doxorubicin, cisplatin, ifosfamide, cyclophosphamide, etoposide, or gemcitabine [18,19].

While patient tolerance to chemotherapy drugs and tissue loss post-surgery are concerns, this can potentially be addressed simultaneously with drug-loaded hydrogels directly targeting the affected osteosarcoma site [20]. In tissue engineering, hydrogels and polymeric implants or scaffolds have been used for their ability to simultaneously release preloaded drugs and induce native tissue regeneration [21,22]. In preclinical osteosarcoma models, hydrogels were studied for their capacity to reduce tumor size with enhanced drug release. In this review, we describe the use of drug-loaded hydrogels for osteosarcoma therapy and subsequent bone regeneration. Furthermore, in-depth details about the future applications of these implantable scaffolds to effectively treat osteosarcoma in animal models are also discussed.

## 2. Materials and Methods

**Figure 1:**
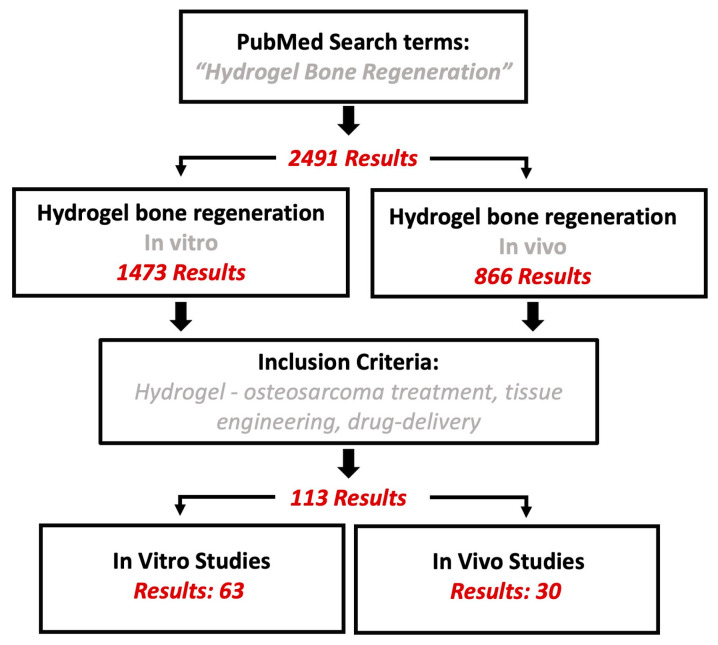
Flowchart of literature search using PubMed.

PubMed was queried for articles with the search term “Hydrogel Bone Regeneration,” which yielded 2491 results. Of the 2491 hits, 1473 articles were studies conducted in vitro, and 866 articles were conducted in vivo. The search was further restricted to the terms “Hydrogel-osteosarcoma treatment,” “tissue engineering,” and “drug-delivery,” resulting in 113 results, of which 63 were in vitro studies and 30 in vivo studies (Figure 1). Herein, we summarize the results of these studies.

## 3. Potential Therapies for Osteosarcoma

The current etiology of osteosarcoma is unknown; however, it is associated with syndromes linked to tumor suppressor gene mutations such as Li-Fraumeni syndrome (p53 mutation), retinoblastoma (Rb mutation), and multiple genetic mutations [14,23,24]. Additionally, exposure to radiation is a risk factor for developing osteosarcoma [1]. Clinically, patients often present with localized bone pain, swelling, and limited range of motion in the affected region. Rarely, pathologic fractures may be present due to osteolytic tumor formation [25,26]. The diagnosis is made through gross imaging studies, such as X-rays, to detect bony tumor formation. However, small tumors are likely not detected through imaging modalities [27]. Recently, advances have been made in the detection of osteosarcoma through the use of nanomaterials in a preclinical setting. Radiolabeled nanomaterials are used to mark tumor cells which in turn increases imaging intensity [28]. Non-invasive imaging through fluorescence dyes attached to nanoparticles can target osteosarcoma and metastasis in mice [29]. While the primary treatment of osteosarcoma includes surgery, chemotherapy, and radiation, patients require multidisciplinary care; a team of surgeons, oncologists, physical therapists, and psychiatrists, among others, are necessary for the proper multifactorial treatment of osteosarcoma in order to address the patient’s medical and socioeconomic needs [30]. Despite extensive involvement in patient care and treatment options, outcomes are usually poor for osteosarcoma, and the exploration of novel treatment options is necessary to reduce patient morbidity and mortality.

Targeted therapy and immunotherapy are popular modalities for treating cancers by targeting driver mutations that result in overexpressed genes and have been performed with lung, thyroid, and skin cancers with promising results [31,32,33]. In osteosarcoma, genetic heterogeneity allows for a wide array of molecular targets for targeted therapy and immunotherapy [14]. Inhibitors against PI3K/mTOR have been developed and have shown anti-tumor effects in vitro; however, not all patients carry this mutation. Alternatively, PTEN is a negative regulator of PI3K/mTOR and can serve as a potential target in future trials [34]. Another common mutation is seen in vascular endothelial growth factor (VEGF), a major regulator of angiogenesis and cell growth. VEGF expression is often increased with metastasis. Anti-VEGF drugs have demonstrated anti-tumor activity against osteosarcoma in clinical trials for single tumors and metastasis [35,36,37,38,39]. Future advancements can place a more focused approach on VEGF mutations to greatly reduce treatment time and survivability in these patients. A promising method to target hematological cancers is known as chimeric antigen receptor (CAR) T-cell therapy [40]. This modality utilizes the patient’s own T-cells modified with CARs to directly target antigens expressed on the malignancy. A major limitation is the lack of use in solid malignancies such as osteosarcoma [40]. To date, multiple ongoing clinical trials are being conducted on CAR-T cell therapy for osteosarcoma; however, no published data have been reported [41]. Interestingly, CAR-T cell therapy against GD2, a glycolipid with low expression in normal tissue, is being explored [42]. GD2 is highly expressed in solid tumors, including osteosarcoma, and has been targeted prior as a treatment for neuroblastoma [43]. Additionally, preclinical studies have demonstrated efficacy in tumor death with GD2-targeted CAR-T cells [42,44]. As a result, CAR-T cell therapy is a viable option for osteosarcoma treatment once further research is conducted.

## 4. Tissue Engineering for Bioresponsive Hydrogels

### 4.1. Synthesis, Fabrication, and Properties of Hydrogels

Hydrogels are polymeric hydrophilic structures that are capable of absorbing up to thousands of times their dry weight in water without losing their chemical or structural properties [45,46]. Hydrogels are physical gels when the internal networks are connected through molecular interactions or secondary forces, including hydrogen bonding, hydrophobic forces, or ionic forces [45]. Chemical hydrogels have permanent physical bonds since they contain covalently crosslinked internal networks [46]. Chemical hydrogels are generated by transforming hydrophobic polymers into hydrophilic polymers and crosslinking them to form a network [45]. Interestingly, physical hydrogels are less stable due to their dependence on external stimuli such as pH, temperature, and ionic strength. On the contrary, chemical hydrogels are less dependent on external stimuli and have a tendency for greater mechanical resistance [46]. Finally, the swelling of hydrogels reaches a limit once the cohesive and osmotic forces reach equilibrium [47,48].

In the preparation of a physical gel, polymer selection depends on two important criteria: (1) a large amount of water molecules must be held within the hydrogel, and (2) the interactions between the polymer chains must be strong enough to form a semi-permanent connection in the molecular network [49]. The forces involved in physical gel formation include hydrogen bonding, hydrophobic forces, and electrostatic interactions between polymer chains [50]. Physically crosslinked hydrogels are assembled without the use of chemical modifications or crosslinking materials [49]. For example, alginate can be crosslinked using divalent cations within a solution [48]. However, despite these advantages, physical hydrogels have inconsistent performance in vivo due to their inflexibility towards gel pore size, chemical functionalization, and degradation [49].

Chemically crosslinked hydrogels allow for drug release through diffusion without dissolution. Chemical crosslinking produces a permanent hydrogel through covalent bonding to connect the polymers [49]. The formation of crosslinks can be performed by the introduction of small cross-linker molecules, photosensitive agents, or by an enzyme-catalyzed reaction. The simplest form of crosslinking occurs between amino groups and aldehydes, forming a Schiff base [51]. In particular, glutaraldehyde can form imine bonds with the amino groups of chitosan through a Schiff reaction [52]. However, a disadvantage of small-molecule crosslinking methods is the possible toxicity of unreacted cross-linker agents in vivo. Photo crosslinking formation depends on the existence of photosensitive functional groups. Conjugating a photosensitive functional group to a polymer allows it to create crosslinks after UV irradiation. For example, azide groups can be incorporated into a polymeric chain of chitosan to create a photo-crosslinked chitosan hydrogel [49]. After UV exposure, the azide groups convert to nitrene groups that are capable of binding free amino groups present in chitosan and lead to in situ formation of a hydrogel within a minute [53]. Photo crosslinking also allows for the rapid formation of hydrogels at a low cost compared to chemical methods that generally require the expensive additions of initiators and/or catalysts [49,54]. However, a disadvantage of this technique is the requirement of prolonged irradiation, which can result in a local rise in temperature that can damage nearby cells and tissue [55]. Finally, enzymatic crosslinking is another approach to linking polymer chains to develop in situ hydrogels. Under physiological conditions, injectable hydrogels were developed by enzymatically crosslinking poly(L-glutamic acid) grafted with tyramine and poly(ethylene glycol), which was shown to have biomedical applications in tissue engineering scaffolds and drug delivery [56]. Advantages of this approach are that enzymatic-cross linked hydrogels are substrate specific, dynamic, and can be applied to controlled drug release systems [57]. Additionally, enzymatic reactions catalyzed by most enzymes occur at neutral pH, moderate temperatures, and aqueous environments, demonstrating applications for in situ formation of hydrogels. Disadvantages of this approach are limited mechanical properties of the gels, instability of some of the enzyme types such as transglutaminases, and few studies conducted utilizing enzymatically-cross linked hydrogels in vivo [57].

Hydrogels may be fabricated through a variety of methods. Novel methods for rapid and efficient production include using 3D printers to create 3D scaffolds for in vitro or in vivo testing. Most importantly, bioprinters can be loaded with different bioinks containing the biomaterial for fabrication [58]. A key benefit of this method is the encapsulation of cells within the bioink prior to printing, thus avoiding the variability in cell seeding in post-printed constructs [48]. Common printing methods include extrusion, inkjet, stereolithography, and laser printing. Each method carries benefits and risks to encapsulated cells in the bioink during the printing process. For example, extrusion printing exposes cells to high-pressure gradients resulting in the lysis of cells during printing. However, this process can print large concentrations of cells. In contrast, laser printing demonstrates no genotoxicity during printing; however, it is an expensive device with complex usage [59]. Overall, the rapid production of hydrogels through 3D printing provides a route for reproducible scaffolds for basic science testing.

### 4.2. Biomedical Applications of Various Hydrogels

Hydrogels have a wide range of biomedical applications as they are biocompatible and biomimetic. The biocompatibility of hydrogels comes from the lack of immunogenic antigens to stimulate a local immune response. The biomimetic capability replicates the extracellular matrix (ECM) with the structure and composition of the gel [60]. Hydrogels can be engineered to control molecular responses, cellular attachments, structural integrity, biocompatibility, and biodegradability. An ideal hydrogel scaffold should mimic the properties of the native tissue’s ECM, allow cell attachments, cell migration, and nutrient diffusion, and induce nearby cells to modify cell behavior [60]. To create bio-responsive scaffolds, researchers have developed synthetic and natural hydrogels that contain a high surface area to volume ratio that mimics the natural tissue micro and macroenvironment [61].

A common natural material used in hydrogels is collagen, the most abundant animal ECM protein. Collagen fibers are triple-stranded helical structures bounded by covalent bonds and hydrogens bonds rendering the fibrils to be self-aggregating [60]. Collagen is an ideal material for hydrogels as it is a natural ECM protein and contains binding sites to encourage cell adhesion and function. Collagen is degraded naturally by serine proteases and collagenases, allowing locally controlled degradation and ECM remodeling by the cells present in the target tissue [62]. Electrospinning, a viable technique used for generating homogenous ultra-thin fibers [60], generates well-aligned and biocompatible collagen fibers. Electrospinning is necessary to create hydrogels with high surface-area-to-volume ratios and porosity to mimic the ECM [63,64]. The heat and low pressure of electrospinning result in weak denaturation, allowing crosslinks between collagen fibers to form and thus increase biocompatibility [60]. Gelatin, a denatured form of collagen, is another natural material used in hydrogels as it retains important bioactive properties such as RGD sequences and metalloproteinase degradation sites [65]. Gelatin is an attractive polymer for tissue engineering due to its ease of modification and low levels of cytotoxicity and immunogenicity [66]. The biocompatibility of gelatin and gelatin-based biomaterials was improved by incorporating a surface-modified hydroxyapatite sponge scaffold and using an alternating electrospinning and soaking process [60]. The electrospinning and soaking processes are used to generate gelatin hydrogels that contain the correct porosity needed for biocompatibility. Additionally, the alternate soaking process was found to promote better cellular proliferation and adhesion [60].

Synthetic polymers such as poly(lactic acid), poly(glycolic acid), poly(ethylene oxide), and poly(ethylene glycol) offer scientists versatile materials with chemical and physical properties that can be altered. Poly(lactic acid) and poly(glycolic acid) are some of the most studied synthetic polyesters that have been shown to have satisfactory biocompatibility and are FDA approved for specific human clinical applications [60,64]. Recently, nano-hydroxyapatite/Poly(lactic acid) scaffolds have been found to be biocompatible with MG-63 cells and derived human bone marrow mesenchymal stem cells [67,68]. Poly(ethylene oxide) and poly(ethylene glycol) are also FDA-approved and are some of the most commonly used polymers utilized in hydrogel synthesis for tissue engineering [60]. Both poly(ethylene oxide) and poly(ethylene glycol) have adjustable mechanical properties that make them excellent scaffolds for producing 3D templates for tissue regeneration [69]. Overall, synthetic blends and polymers are easier to process than naturally derived polymers and have more predictable results. However, synthetic polymers are not as bioactive or biocompatible as naturally derived polymers [70].

**Figure 2:**
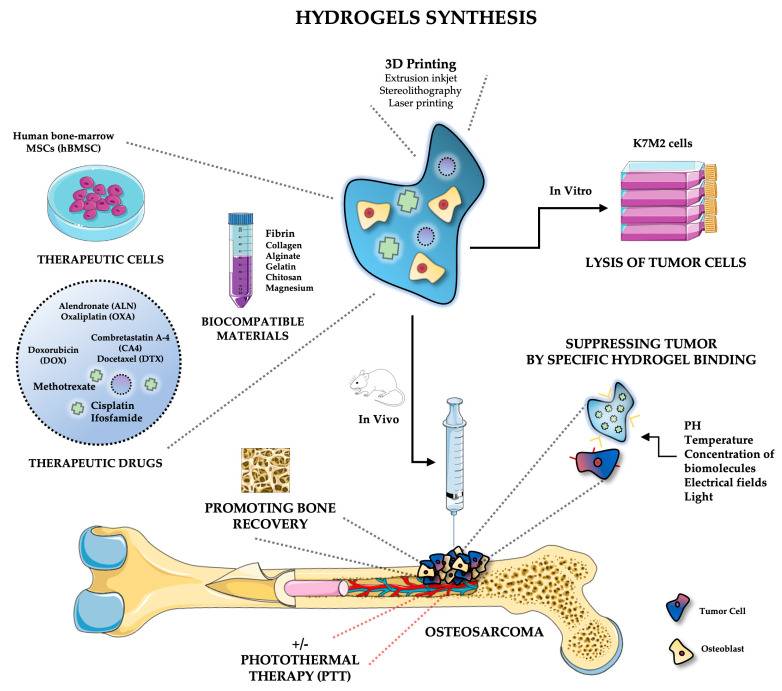
Example schematic of hydrogel fabrication and site-specific incorporation to treat osteosarcoma. Hydrogels may be stimuli-responsive by self-regulation in response to pH, temperature, or mechanical stress. The hydrogels may also include properties that are pro-osteogenic while suppressing or even reducing tumor growth. This figure was generated using the Servier Medical Art. Servier Medical Art is licensed under a Creative Commons Attribution 3.0 Unported License [76].

Stimuli-responsive hydrogels, or “smart” hydrogels, are used to stimulate drug release based on external triggers such as temperature, pH, the concentration of biomolecules, electrical fields, and light [71]. Smart hydrogels respond to triggers in a manner that is predictable, reversible, intensity-scalable, and reproducible [72]. Additionally, stimuli-responsive hydrogels have the capability of returning to their original shape after the trigger is withdrawn [73]. Smart hydrogels have many advantages in drug delivery systems as they reduce dosing frequencies, maintain the correct therapeutic concentration in a single dose, and minimize drug side effects [74]. For example, pH-responsive hydrogels, which are high molecular polymers, undergo volume or phase transitions when the pH of the external medium is altered. pH-responsive hydrogels are useful because certain injuries, such as wounds and inflammation, cause pH variations that can be exploited for targeted drug delivery to specific tissues and organs [72]. Another example of a smart hydrogel is a temperature-responsive hydrogel that changes shape, volume, or size due to changes in physiological temperatures [75]. Most utilized temperature-responsive hydrogels are liquid or semi-solid at ambient temperatures and undergo a solid-to-gel transition at body temperature. This characteristic allows scientists to load a therapeutic compound onto a hydrogel in the liquid state and then easily solidify and administer it upon application. Through these advancements in tissue engineering, hydrogels are able to be developed to target osteosarcoma to induce tumor cell death and increase survivability (Figure 2).

## 5. Hydrogels for Osteosarcoma Therapy

### 5.1. Drug Delivery

Combination drug therapies are commonly administered to induce cancer cell death in osteosarcoma. However, only a small portion of intravenously administered drugs can reach their organ target in vivo, while the high dose can damage surrounding tissues and result in toxicity [77]. Further, neurotoxicity may also occur due to drug penetration of the blood-brain barrier leading to increased adverse events. These off-site adverse effects reduce the efficacy and bioavailability of existing chemotherapeutic agents [78]. Due to their extensive 3D polymerized structure, hydrogels are capable of being effective drug depositories to allow for local drug delivery with the ability to respond to exogenous and endogenous triggers. The use of hydrogels as a repository for drug delivery is an approach that has gained attention in the last decade as it proves to be an effective targeted therapy for localized pathology [77,79]. Additionally, hydrogels have the capability of being integrated with other drug delivery systems, such as liposomes or microspheres, which synergistically allows for better performance [80]. For example, Wu et al. (2018), photo crosslinked gemcitabine (GEM) hydrochloride loaded liposomes with a gelatin methacryloyl (GelMA) hydrogel to test its efficacy for osteosarcoma ablation [81]. The authors observed a controlled and sustained release of GEM from the hydrogel that lasted 4 days in vitro. Additionally, the hydrogel demonstrated the ability to inhibit osteosarcoma in vivo (BALB/c MG-63 bearing mice) [81].

**Figure 3:**
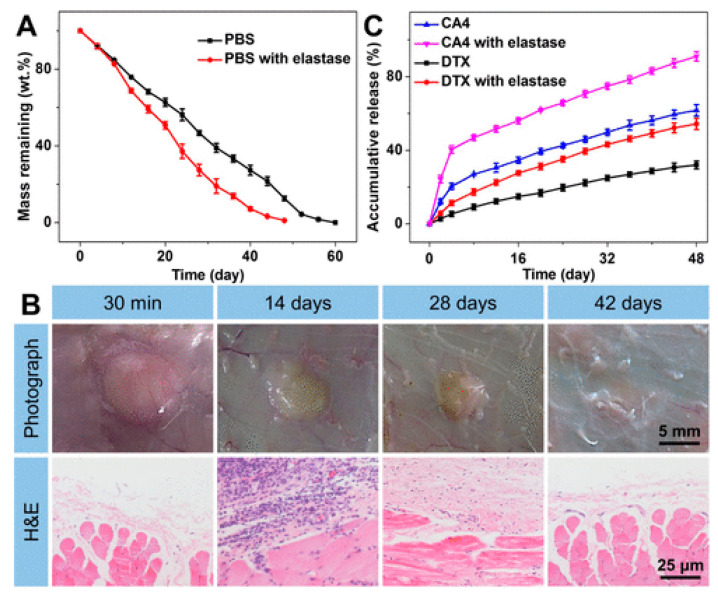
(**A**) Scaffold degradation over 60 days with and without elastase. (**B**) Biocompatibility was assessed through subcutaneous injection of the gel, followed by histological analysis. (**C**) Release of DTX and CA4 from the hydrogels over 48 days. Reprinted with permission from [82].

Due to the diversity of tumor pathogenesis, the effect of a single chemotherapeutic drug may not be successful and synergistic chemotherapy is required to address this issue. A recent study by Zheng et al. (2017) loaded combretastatin A-4 (CA4) and docetaxel (DTX) into a thermosensitive injectable polypeptide hydrogel and reported the preferential release of CA4 in 80 eight-week-old BALB/c male mice with K7 osteosarcoma cells [82]. CA4 can disrupt blood vessel structure in tumors by binding endothelial cell tubulin and blocking tumor necrosis by inhibiting the exchange of oxygen and other nutrients [83]. The sustained release of DTX led to the destruction of tumor surface cells, resulting in apoptosis. The co-loading of both CA4 and DTX resulted in a significantly smallest tumor volume change compared to the control and the largest necrotic area of ~90.5%. Histological analyses of internal organs (heart, liver, spleen, lung, kidney) indicated no toxicity of the dual-loaded hydrogel [82]. Additionally, the in vitro release of CA4 and DTX indicated that within 48 days, 54% of the loaded DTX was released from the co-loaded hydrogel with no burst release observed (Figure 3). The constant release observed of DTX ensured that the effective drug concentration was met in situ, which avoided significant side effects. However, within the same time period, 90% of CA4 was released from the co-loaded gel. The scientists noted a slight initial burst release during the first 8 days as a result of its quick diffusion from the co-loaded gel surface [82].

Similarly, Sun et al. (2020) co-loaded Alendronate (ALN) and Oxaliplatin (OXA) onto a mPEG45-PLV19 thermosensitive hydrogel [84]. OXA is a widely used anti-tumor drug that contains platinum atoms that crosslink DNA to antagonize transcription and replication [85]. ALN is a bisphosphonate, an osteoclast inhibitor, that can target bone tumors based on its affinity for hydroxyapatite and inhibit bone destruction [86]. To establish osteosarcoma, the authors injected K7M2 osteosarcoma cells into the medullary cavity of the tibia of 20 four-week-old BALB/c female mice. The mice injected with the ALN-OXA dual-loaded hydrogel system possessed the smallest dissected tumor (weight 2.81 ± 0.26 g) compared to the other experimental groups. Additionally, the ALN-OXA group had a 60.4% tumor suppression rate, achieving the most statistically significant tumor inhibition effect. The degradation of the ALN-OXA hydrogel was found to be stable and with a degradation rate of 50% reached in approximately 27 days. Additionally, the OXA-ALN-loaded hydrogel was found to slowly release 40% of the loaded drugs within 15 days. Furthermore, histological analyses of the heart, liver, spleen, and kidneys showed no noticeable morphological changes in the osteosarcoma-bearing mice given the ALN-OXA hydrogel compared to the control group [84]. These findings clearly indicate that the co-loaded ALN-OXA hydrogel can inhibit osteosarcoma progression while limiting the toxic effects of chemotherapy.

Another study conducted by Tan et al. (2021) used Curcumin (Cur) microspheres loaded in hybrid methylcellulose hydrogels to target K7M2wt osteosarcoma cells [87]. Delayed release of Cur was observed in the hydrogels that were sustained for up to 14 days. With laser irradiation, Cur released from the hydrogels was nearly 30% within 2 days. However, there was nearly 15% release without irradiation in the same period. The slow release is a promising factor for the long-term treatment of osteosarcoma. Anti-tumor capability against K7M2wt cells was demonstrated through nearly a 75% reduction in viable cells after exposure to Cur and irradiation. There was a 50% reduction in viability when only exposed to irradiation and the hydrogel without Cur. Tumor ablation was assessed in vivo through 20 tumor-bearing female BALB/c mice. After treatment, the tumor size was the smallest (~0.5 g) in mice treated with Cur and irradiation [87]. These findings show that using Cur and irradiation is a potent combination to eradicate osteosarcoma cells in vitro and in vivo. Similarly, Ma et al. (2015) loaded doxorubicin, cisplatin, and methotrexate into a thermosensitive PLGA-PEG-PLGA hydrogel that demonstrated cytotoxicity against Saos-2 and MG-63 osteosarcoma cells in vitro and Saos-2 xenografts in vivo. The PLGA-PEG-PLGA copolymer had no cytotoxic effects against the tumor cell lines; however, the addition of the chemotherapeutic agents had a synergistic anti-tumor effect. The triple drug combination hydrogel had approximately an 80% reduction in viability in Saos-2 and MG-63 cells. This was supported by an upregulation in BAX and caspase-3 and a downregulation in Bcl-2. Additionally, the triple drug combination had the lowest tumor volume and weight in vivo when assessed in 6 male BALB/c mice. Drug release of nearly 80% of the loaded drugs was observed over 11 days in vitro; however, tumor suppression was observed for up to 16 days in vivo [88].

### 5.2. Bone Regeneration

The post-surgical defect present after the resection of osteosarcoma is often large and difficult to heal. Osteogenesis in in vivo models can be stimulated through the addition of scaffolds, growth factors, stem cells, and other small molecules [89]. Hydrogels can target tumor remnants in bony defects and encourage natural bone regeneration at the site of injury due to their biocompatibility, bioactivity, biodegradability, and osteoinductive properties [90]. Biomimetic hydrogels strongly resemble the ECM and therefore provide the necessary support for mesenchymal stem cells (MSC) to proliferate and differentiate into osteoblasts and ultimately regenerate lost bone [91,92].

Hydrogels have also been developed that rapidly induce bone healing, limiting the necessity of bone grafts. Zhang et al. (2018) emphasized the importance of glucocorticoids and magnesium cofactors as they induced MSCs toward osteogenic differentiation [93]. A nanocomposite hydrogel of hyaluronic acid (HA) and pamidronate (PAM) carried encapsulated human MSCs, dexamethasone phosphorylate (DexP), and magnesium cofactor to the site of bone injury to induce a positive feedback loop of bone regeneration. The Mg2+ released from the hydrogel-activated alkaline phosphatase (ALP) dephosphorylated the DexP, activating it. This accelerated the release of dexamethasone and increased osteogenesis. To test the efficacy of the hydrogel in vivo, femoral bone defects were surgically created in skeletally mature female New Zealand white rabbits, and after 8 weeks post-hydrogel implantation, the bone volume/tissue volume (BV/TV) showed a 20% significant increase with the HA-PAM-Mg-DexP hydrogel [93]. Gene expression analysis demonstrated an increase in type 1 collagen, osteocalcin, and Runx2 with the HA-PAM-Mg-DexP hydrogel. On day 7, the expression level of type 1 collagen was 2.7 compared to the control, which had 1.5. The expression of osteocalcin and Runx2 also significantly increased in comparison to the control (2.2 vs. 1, respectively).

**Figure 4:**
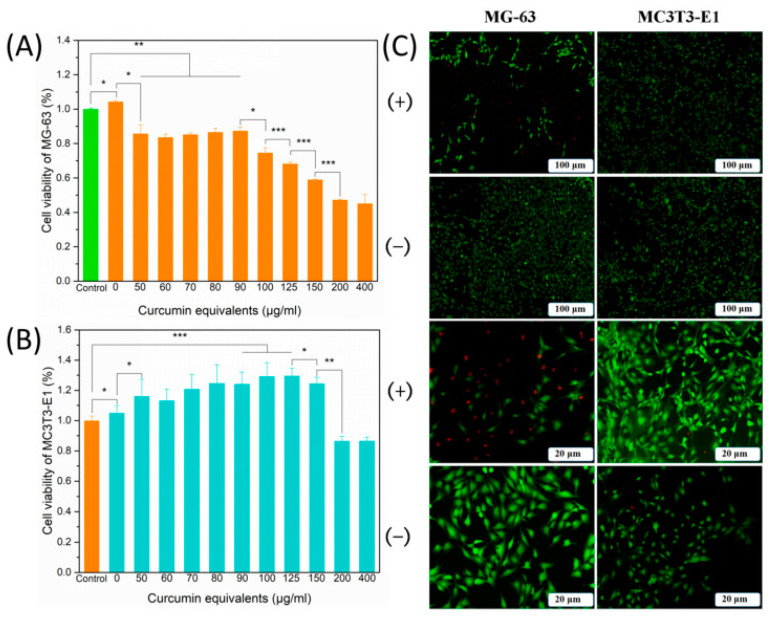
(**A**) Cell viability of MG-63 osteosarcoma cells when exposed to curcumin (Cur). (**B**) Cell viability of MC3T3-E1 pre-osteoblasts when exposed to Cur. (**C**) Live and dead images of MG-63 and MC3T3-E1 cells exposed to curcumin-loaded scaffolds (+) and without (−). * *p* < 0.05, ** *p* < 0.01, *** *p* < 0.001. Reprinted with permission from [94].

The development of hydrogels capable of targeting residual osteosarcoma cells while encouraging non-tumor cell growth is essential for a dual-targeted system for treatment. Yu et al. (2021) demonstrated the development of Cur-loaded chitosan nanoparticles encapsulated in a methacrylated silk fibroin/hyaluronic acid (CCNPs-SF/HAMA) hydrogel, which proved to be effective in reducing the viability of osteosarcoma cells and promoting the proliferation of osteoblasts in vitro [94]. Cur, a natural polyphenol derived from turmeric Curcuma longa, has shown anti-tumor, anti-oxidant, and anti-inflammatory activities against osteosarcoma, as well as increased osteoblast proliferation and bone formation [95,96,97]. The CCNPs-SF/HAMA hydrogels loaded with Cur demonstrated that 150 μg/mL of Cur demonstrated simultaneous anti-cancer effects in MG-63 cell cultures, significantly decreasing cell viability from 100% to approximately 56% and osteoblast proliferative effects in MC3T3-E1 cell cultures, significantly increasing the cell viability from 100% to 124.5% (Figure 4) [98].

**Figure 5:**
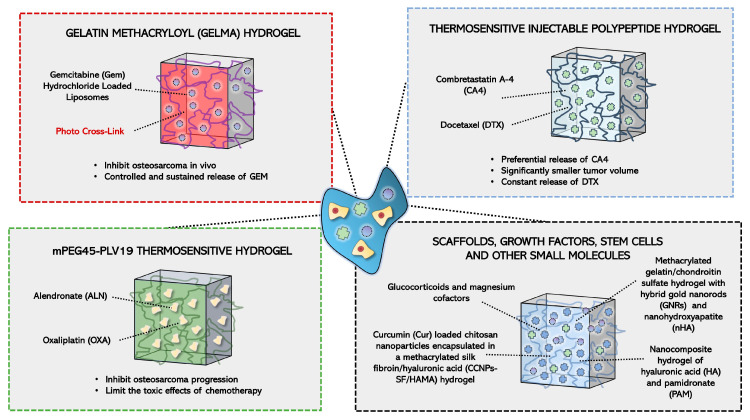
Schematic of hydrogel combination drug therapies, commonly administered to induce slow drug release and cancer cell death in osteosarcoma [81,82,84,92,93,94]. This figure was generated using the Servier Medical Art. Servier Medical Art is licensed under a Creative Commons Attribution 3.0 Unported License [76].

Liao et al. (2021) fabricated a methacrylated gelatin/chondroitin sulfate hydrogel with hybrid gold nanorods (GNRs) and nanohydroxyapatite (nHA) and used photothermal therapy (PTT) to eradicate the tumor remnants while simultaneously depositing MSCs that encouraged bone growth. The eradication of the tumor composed of K7M2 cells was tested in vitro [92]. PTT was performed using multiple power densities, and a significant decrease in tumor cells was noted using 0.99 W/cm3 power density as the tumor cell viability dropped from 100% to 1.2%. In an in vivo demonstration, the experimental group (surgery + hydrogel + PTT) successfully eliminated tumor remnants using a 0.99 W/cm^3^ power density for the PTT. Additionally, the authors showed a significant decrease in tumor volume from approximately 2500 mm^3^ in the control group to 50 mm^3^ in the experimental group, with no further recurrence of the osteosarcoma. Additionally, a significant increase in bone volume was observed with microCT and H & E staining, measuring from 3.1 mm^3^ in the control group up to 5.4 mm^3^ in the surgery + hydrogel + PTT group [92]. A summary of the studies used to treat osteosarcoma are detailed in Table 1. A schematic of hydrogels of varying composition and loaded drugs is shown in Figure 5.

## 6. Non-Hydrogel Scaffolds for Osteosarcoma Therapy

**Figure 6:**
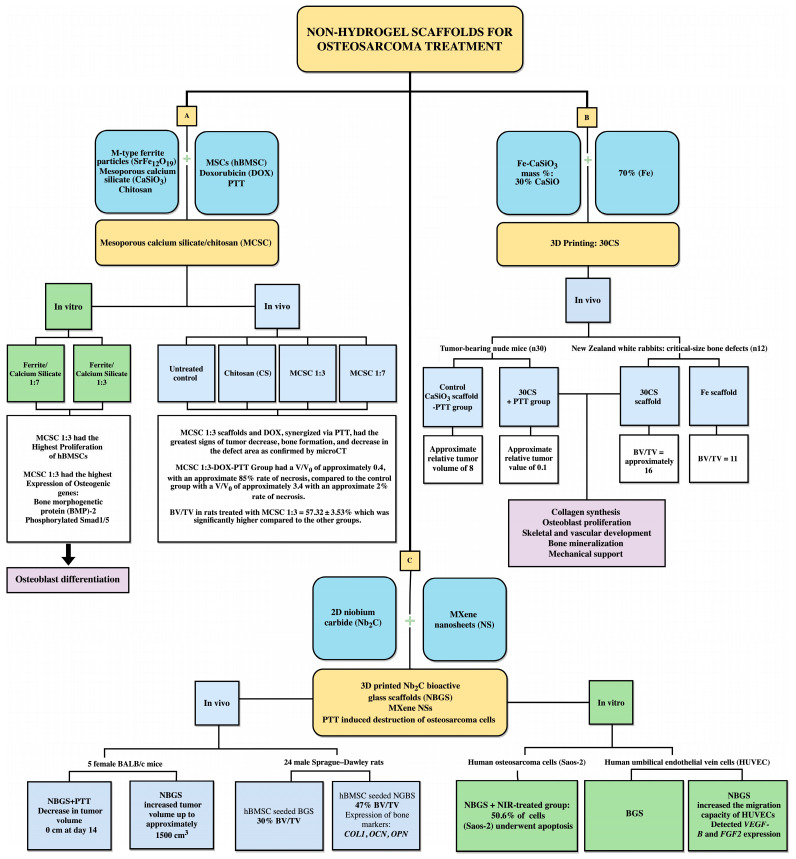
Schematic overview of some preclinical studies on the use of non-hydrogel scaffolds for the treatment of osteosarcoma [99,103,109].

While the use of hydrogels has shown success in the treatment of osteosarcoma and regeneration of bone defects, there also has been much research in the development of non-hydrogel scaffolds for osteosarcoma therapy [98]. Yang et al. (2018) worked on establishing a bifunctional scaffold, one that could treat post-resection osteosarcoma remnants via drug (doxorubicin) release and PTT while simultaneously encouraging bone regeneration in the defect using human bone-marrow MSCs (hBMSC) [99]. A mesoporous calcium silicate/chitosan (MCSC) scaffold consisting of M-type ferrite particles (SrFe12O19), mesoporous calcium silicate (CaSiO3), and chitosan was fabricated. SrFe12O19 enhanced the PTT efficacy using doxorubicin (DOX) as the chemotherapeutic agent [100]. The MCSC scaffolds were developed with two different ferrite/calcium silicate ratios: 1:7 and 1:3. hBMSCs demonstrated the highest proliferation on the MCSC 1:3 scaffolds and had the highest expression of osteogenic genes, bone morphogenetic protein (BMP)-2, phosphorylated Smad1/5 and Runx2, all of which indicate osteoblast differentiation [101,102]. Further, an in vivo experiment was conducted on rat bone defect models with four experimental groups: untreated control, chitosan (CS), MCSC 1:3, and MCSC 1:7 treated. The experimental group treated with MCSC 1:3 scaffolds and DOX, synergized via PTT, had the greatest signs of tumor decrease, bone formation, and decrease in the defect area, as confirmed by microCT. The relative tumor volume was determined by a ratio at day 12 (V) and day 0 (V0). The group treated with MCSC 1:3-DOX-PTT had a V/V0 of approximately 0.4, with an approximate 85% rate of necrosis, compared to the control group with a V/V0 of approximately 3.4 with an approximate 2% rate of necrosis. The BV/TV in rats treated with MCSC 1:3 scaffolds was 57.32  ±  3.53%, which was significantly higher compared to the other groups (Figure 6) [99].

Ma et al. (2018) designed a Fe-CaSiO3 (mass %: 30% CaSiO3 and 70% Fe) compound scaffold, named 30CS, via 3D printing and demonstrated tumor killing and bone regeneration in vivo [103]. The release of Fe ions by the scaffold encourages the production of reactive oxygen species (ROS), which results in tumor cell apoptosis via DNA damage [104]. Further, the scaffolds exhibited synergistic effects with PTT to further eradicate the tumor remnants after resection. The 30CS scaffolds also provided mechanical support for rabbit bone mesenchymal stem cells (rBMSC) and encouraged bone formation in vivo. In 30 tumor-bearing nude mice, the control CaSiO3 scaffold without PTT had an approximate relative tumor volume of 8, whereas the 30CS + PTT group had an approximate relative tumor value of 0.1. The authors further assessed bone regeneration in 12 New Zealand white rabbits with critical-size bone defects. The increase in bone volume was significantly higher, with the 30CS scaffold showing a BV/TV of approximately 16 vs. 11 for the Fe scaffold [103] (Figure 6). Additional studies also showed that Si ions released from these scaffolds stimulated collagen synthesis, osteoblast proliferation, and skeletal and vascular development [105,106]. Similarly, released Ca ions enhanced osteogenesis and bone mineralization [107,108]. Overall, these experiments proved the efficacy of the 30CS scaffold in tumor treatment and bone regeneration.

Another study integrated 2D niobium carbide (Nb2C) and MXene nanosheets (NS) into 3D printed porous bioactive glass scaffolds (BGS) designed with PTT-induced destruction of osteosarcoma cells and bone regeneration by encouraging neovascularization in the defect [109] (Figure 6). Nb2C bioactive glass scaffolds (NBGS) MXene NSs are biocompatible and biodegradable materials due to their intrinsic photoresponse in the second near-infrared (NIR-II) biological window for PTT [110]. The researchers explored the in vitro cytotoxic effects of this scaffold on the human osteosarcoma cell line Saos-2. In the NBGS + NIR-treated group, 50.6% of the tumor cells underwent apoptosis, as determined through immunofluorescence. The same procedure was conducted in vivo with five female BALB/c mice. NBGS + PTT showed the greatest decrease in tumor volume, measuring 0 cm^3^ on day 14, compared to NBGS, showing increased tumor volume up to approximately 1500 cm^3^. After validating the cytotoxicity of the scaffold with PTT on osteosarcoma cells, its capacity to promote angiogenesis and induce osteogenesis was evaluated. It is already well-known that blood vessels and angiogenesis play a pivotal role in osteogenic differentiation and bone healing [111]. The researchers compared the effects of the BGS and NBGS scaffolds in promoting angiogenesis in vitro using human umbilical endothelial vein cells (HUVEC) to confirm the NBGS increased the migration capacity of HUVECs as confirmed by the detection of VEGF-B and FGF2 expression [112]. Additionally, calvarial defects were created in 24 male Sprague–Dawley rats with hBMSC-seeded NBGS and BGS scaffolds to assess bone regeneration. Significant bone regeneration was demonstrated in the NBGS group, with 47% BV/TV, compared to the BGS (30%) group. Bone regeneration was also confirmed via the expression of bone markers, COL1, OCN, and OPN genes [113,114]. Overall, the experiment demonstrated that the NBGS scaffold effectively destroyed tumor cells via PTT and prompted bone regeneration at the site of the bone defect [109].

## 7. Future Applications of Hydrogels Designed for Osteosarcoma Therapy

While hydrogels have shown promising results in treating osteosarcoma, there are still unresolved research areas. Hydrogels are often prone to failure due to poor mechanical and structural stability and are unable to maintain complex structures [115]. Even though most hydrogels are fabricated to mimic the ECM, the lack of mechanical strength prevents them from providing substantial mechanical support to cells and tissues [116]. Natural sources of hydrogels such as collagen, gelatin, alginate, and chitosan are often considered to be good candidates for drug and cell-based therapy because of their biocompatibility but have limited use due to their lack of stability and weak mechanical qualities [117]. Alginate hydrogels are a primary example, as they require an additional crosslinking step to maintain their polymer structure; they also lose mechanical strength within a very short period of time [118]. A larger degree of crosslinking has been connected to a decrease in swelling ratios and an increase in the brittleness of the hydrogels, which may further indicate mechanical instability [117]. Hydrogels have low tensile strength and, as a result, can result in premature disintegration within the targeted site. In the case of drug delivery, the large pore size coupled with the large water content of hydrogels could limit the release of hydrophobic or highly specific homogenous drugs [119].

Despite these drawbacks, hydrogels have many advantageous and potential translational applications. Hydrogels allow for efficient drug delivery because they can be administered through parenteral, nasal, ocular, and topical routes [120]. Additionally, hydrogels make it possible to prevent off-site drug exposure to provide targeted therapy [121]. The site-specific delivery of drugs also allows the target site to receive higher doses of medication at the time of administration, leading to a reduced number of doses necessary to achieve the desired therapeutic effect [121]. Currently, regenerative hydrogels are being used in the United States and European Union to provide pain relief and functional improvement and help tissue regeneration [122].

Despite the translational applications, there are still limitations to using hydrogels in clinical trials. Many hydrogel systems, such as poly(phosphazene), pluronic, and poly(N-isopropylacrylamide), have non-biodegradable and non-biocompatible characteristics that may have unintended or unknown effects [122]. The release of therapeutic agents also presents a challenge for clinical trials. For drugs that require sustained release (such as protein-based drugs), the crosslinking mesh and solute elution cannot be disrupted as the drug will not be properly released at the target site [123]. Injectable hydrogels currently comprise 26% of all clinical trials involving bulk hydrogels, but there is a challenge to create hydrogels with a viscosity low enough to be delivered through a needle and syringe [123]. Additional properties of hydrogels that must be considered for use in clinical trials involve the molecular weight of polymer chains, the density of crosslinking, and the viscosity of the solution.

Even with the possible limitations of hydrogels in clinical trials, they may help to treat osteosarcoma less invasively. Current methods of treatment for osteosarcoma involve surgical resection and adjuvant chemotherapy. Some of the biggest challenges for osteosarcoma treatment are chemoresistance and metastasis prevention [124]. Studies have shown that hydrogels can treat tumors due to their biocompatibility and porous structures [80]. Hydrogels can be loaded with tumor therapy drugs that can be inserted intravenously to replace systemic chemotherapy. In one example, hydrogels loaded with PEG-g-chitosan improved the infiltration of T lymphocytes into the gel and allowed the controlled release of these cells to the tumor site [125]. Often, tumor pathogenesis requires the use of more than one drug, and the current method of administering multiple drugs is synergistic chemotherapy. Currently, there is an effort to produce hydrogel scaffolds with stronger mechanical and biological properties [124]. For osteosarcoma treatment, this entails using materials in the gels that improve the flexibility and structure of the hydrogels [126]. Some materials under consideration are chitosan and magnesium because these are known for their mechanical properties and biocompatibility [126]. Hydroxyapatite is being studied for its ability to promote bone growth and tissue repair, but it has not been frequently used in hydrogels because it is brittle [127]. However, carbon nanofillers such as carbon nanotubes, graphene oxides, and graphene oxide-carbon nanotubes are also being studied to examine if they can reduce the brittle properties of hydroxyapatite [128].

Hydrogels allow targeted drug release and aid in reducing the adverse effects of neoadjuvant chemotherapy and anti-cancer drugs, but inherently hydrogels carry some side effects. Hydrogels made with synthetic polymers such as polylactide (PLA), poly-lactide-co-glycolide (PLGA), polyglycolide (PGA), poly-(D,L-lactic acid) (PDLLA), polycaprolactone (PCL), poly-ethylene-glycol (PEG), poly(vinyl alcohol) (PVA), poly(N-isopropylacrylamide) (PNIPAM), and polyacrylamide (PAM) can induce an immune response in the body or expose toxicity to the cells [118]. In addition, many of these polymeric-based biomaterials cannot be incorporated into host tissues and are thus toxic foreign materials that must be degraded in the body. Hydrogels made with natural polymers do not have the same problems and do not cause immune or toxic reactions [118]. Thus, there is an emphasis on using natural hydrogels because of their non-toxic, biocompatible, and biodegradable properties. There are fewer long-term side effects caused by hydrogels because of their ability to degrade in the body after the release of the desired drug(s) [129].

Due to the convenience of 3D printing, the synthesis and fabrication of hydrogels are both cost-effective and time-efficient [59,130]. 3D printing also allows for complex crosslinking and structure formation without requiring long periods of time and costly materials. In addition, hydrogels are generally composed of easily accessible compounds such as hyaluronic acid, alginate, fibrin, collagen, gelatin, and chitosan, which also help to reduce overall production costs [59]. The major technical drawback of fabricating hydrogels is creating a stable structure despite the poor mechanical properties of most hydrogels [131]. Further research is needed to determine the best structure and material to use for maximizing the therapeutic potential of hydrogels, especially for osteosarcoma.

## 8. Hydrogel Use in Clinical Trials on Osteosarcoma

Examining clinical trial registries is fundamental for acquiring an approximate overview of the progress of ongoing research efforts. Our group performed a search in the National Institutes of Health’s (NIH) ClinicalTrials.gov registry (access dates up to and including 1 March 2023) for all registered clinical trials, using the following keywords: “hydrogels” and “cancer treatment.” No exemption criteria were applied to filter the initial results, and the search included both studies marked as completed and as actively enrolling. The search brought up 57 clinical trials in the area of hydrogels used in various cancer therapy studies, mostly located in Europe (16) and the United States (24). There were 2 phase 1 clinical trials, 11 phase 2 trials, 14 phase 3 trials, and 2 phase 4 clinical trials. The search included 5 studies based on a young population (0–17), 10 studies based on adults (18–64), and the remaining 42 studies focused on subjects 65 and older. However, most of the above-mentioned clinical trials investigate therapeutic options for conditions such as gastrointestinal cancer, carcinoma, or male genital cancers. None of the queried studies partake in the topic of bone malignancies such as osteosarcoma.

We also searched with the keywords “hydrogel” and “bone recovery”, with no exception criteria included. The search resulted in 19 completed or actively enrolling clinical trials that exclusively target the adult population. Overall, the clinical trials focus on therapeutic methods for conditions such as bone resorption, tooth loss, periodontitis, chondral defect, or articular cartilage defect, with no significant mention of bone malignancies. Despite the many preclinical studies investigating localized hydrogel therapy for osteosarcoma treatment as a means to replace systemic chemotherapy, the need for highly translational trials remains unmet. Further, the reduced number of ongoing clinical trials proves a significant gap between the successful implementation of a preclinical strategy that can translate into the rigor, limitations, and regulations of clinical trials.

## 9. Conclusions

While numerous advancements have been made clinically for treating and managing osteosarcoma, this tumor remains one of the most devastating bone cancers. Surgical management with chemotherapy has proven to be effective in preventing the recurrence of osteosarcoma; however, patient responses vary. The treatment of osteosarcoma with non-surgical management has been explored both in vitro and in vivo through the use of hydrogels loaded with chemotherapeutic drugs to assess tumor regression and subsequent bone regeneration. Synergistic scaffolds that can display cancer cytotoxicity while promoting bone regeneration of the defect are superior in treatment due to their dual-targeted approach. However, such biomaterials have not yet reached the market and are still performed in laboratory testing to fully assess the side-effect profile and to optimize successful functional parameters. The future applications of hydrogels for osteosarcoma treatment should emphasize in vivo revascularization of bone defects and stimulate neo-osteogenesis from post-surgical resection or direct chemotherapy-targeted osteosarcoma regression. Currently, there is a lack of data supporting increased neovascularization of bone, while there is data supporting bone formation. Furthermore, advancements in hydrogel-based therapy with novel stimuli-responsive hydrogels can pose a breakthrough in potential treatments that can be adjusted for tumor microenvironments to rapidly eradicate osteosarcoma cells without many side effects. Lastly, clinical trials are needed to potentially address the translational applications of the hydrogel’s dual-targeted approach.

## Figures and Tables

**Table 1:** Summary of studies treating osteosarcoma.

Author/Year	Experiment	Strain	Hydrogel Composition	Benefits	Drawbacks	Methods	Results
Wu et al., 2018 [80]	In vivo, in vitro	MG-63 cells, BALB/c mice injected with MG-63 cells (*n* = N/A)	Gemcitabine (GEM) loaded liposomes with gelatin methacryloyl (GelMA)	Rapid cell death within 4 h, sustained release of gemcitabine	Inflammatory reaction in vivo, low cell death in vivo	Live/dead, degradation %, drug release %, neutrophil counting, H & E staining	GEM-GelMA scaffolds demonstrated cytotoxicity in vitro but marginal tumor suppression in vivo. An inflammatory reaction was present in vivo.
Zheng et al., 2017 [81]	In vivo, in vitro	BALB/c male mice injected with K7 cells (*n* = 80)	Poly(L-alanine-co-L-phenylalanine)-block-poly(ethylene glycol)-co-poly(L-alanine-co-L-phenylalanine)) hydrogel co-loaded with combretastatin A-4 and docetaxel	Biocompatible, biodegradable, rapid release and diffusion of drugs, apoptosis of osteosarcoma, blockage of cell proliferation	Safety concerns due to rapid weight loss, accelerated diffusion rate within 1 week may risk drug overloading	Degradation %, drug release %, H & E staining, tumor volume measurements, the survival rate	CA4-DTX co-loaded hydrogels caused a reduction in osteosarcoma tumor size and did not display any adverse effects on any internal organs. The hydrogel demonstrated sustained release over 48 days of DTX.
Sun et al., 2020 [83]	In vivo, in vitro	BALB/c female mice injected with K7M2 cells (*n* = 20)	mPEG45–PLV19 co-loaded with oxaliplatin and alendronate	Decrease in tumor weight, tumor suppression, no organ toxicity, biocompatible	Safety concerns due to rapid weight loss, the burst effect of drug release may influence temperature-sensitive hydrogel	MTT assay, degradation %, drug release %, tumor volume measurements, H & E stain, microCT	ALN-OXA-loaded hydrogels reduced tumor size and growth. The hydrogel degraded by 50% within 27 days and released 40% of the drugs within 15 days. No toxicity was shown to the internal organs of the mice.
Tan et al., 2021 [86]	In vivo, in vitro	NIH3T3 cells, K7M2wt cells, BALB/c female mice (*n* = 24)	Curcumin-loaded PLGA microspheres in methylcellulose and IR820	Biodegradable, biocompatible, bone regrowth	Necessary use of irradiation with Cur for optimal effects, inflammatory reaction in vivo	Live/dead, CCK-8 viability assay, H & E stain, microCT, tumor volume measurements	Cur-loaded PLGA/Methylcellulose scaffolds demonstrated cell cytotoxicity; however, prominent effects were seen with the addition of irradiation. Tumor suppression was seen in vivo.
Ma et al., 2015 [87]	In vivo, in vitro	Saos-2 cells, MG-63 cells, BALB/c male mice with Saos-2 xenografts (*n* = 48)	PLGA-PEG-PLGA triblock copolymer loaded with doxorubicin, cisplatin, and methotrexate	Slow release, pro-apoptotic, no organ toxicity	PLGA-PEG-PLGA copolymer has minimal anti-cancer effects alone. Bone regeneration was not assessed	Drug release %, MTT assay, PCR, tumor volume measurements, H & E stain	The addition of chemotherapeutic drugs is necessary for cytotoxicity. Tumor volume and cell viability were suppressed. No toxicity was shown to the internal organs of the mice.
Liao et al., 2021 [91]	in vivo, in vitro	K7M2 cells, Mice MSCs, and BALB/c mice for the in vivo assessment (*n* = 20)	Methacrylated gelatin/methacrylated chondroitin sulfate hydrogel with hybrid gold nanorods (GNRs) and nanohydroxyapatite (nHA)	Decrease in osteosarcoma and increased deposition of stem cells for dual-purpose treatment, minimal destruction of healthy tissue.	Potential inflammatory response due to macrophage and lymphocyte infiltration, bone grew for 2 weeks and was not assessed for longer.	Live/dead, H & E stain, microCT, bone volume measurements	The methacrylated gelatin/methacrylated chondroitin sulfate hydrogel with hybrid gold nanorods (GNRs) and nanohydroxyapatite (nHA) eliminated the tumor remnants with no further recurrence of the osteosarcoma. An increase in bone volume was seen.
Zhang et al., 2018 [92]	in vivo, in vitro	Human MSCs, female New Zealand white rabbits (*n* = 3)	Hyaluronic acid (HA), pamidronate (PAM), Mg^2+^ cofactor, and dexamethasone phosphorylate (DexP)	Flexible hydrogel with a non-restricted microenvironment for cells, quick molding to bone defect, slow degradation with steady, sustained release	Angiogenesis and bone growth were not addressed	Fluorescent staining, PCR, microCT, H & E stain	Hydrogel loaded with human MSCs, dexamethasone, and magnesium showed increased bone regeneration in a rabbit femur defect. Greater bone volume was demonstrated in the treated femur.
Yu et al., 2021 [93]	in vitro	MG-63 and MC3T3-E1 cells	Curcumin-loaded chitosan particles encapsulated in a methacrylated silk fibroin/hyaluronic acid hydrogel	Low immunogenicity, can handle high compression forces, adherence to tissue	No data regarding how the hydrogel would perform in vivo	Drug release %, Live/Dead stain, MTS assay	CCNP-SF/HAMA hydrogels demonstrated anti-tumor activity and osteoblastic proliferation in vitro with varying Cur concentrations.

## Data Availability

The data presented in this study are available on request from the corresponding authors.
